# Extracorporeal Shock Wave Therapy (eSWT) in Spinal Cord Injury—A Narrative Review

**DOI:** 10.3390/jcm13175112

**Published:** 2024-08-28

**Authors:** Józef Opara, Robert Dymarek, Mirosław Sopel, Małgorzata Paprocka-Borowicz

**Affiliations:** 1 Department of Physiotherapy, The Jerzy Kukuczka Academy of Physical Education, 40-065 Katowice, Poland; 2 Department of Physiotherapy, Faculty of Health Sciences, Wroclaw Medical University, 50-367 Wroclaw, Poland; robert.dymarek@umw.edu.pl (R.D.); malgorzata.paprocka-borowicz@umw.edu.pl (M.P.-B.); 3 Faculty of Medicine, Wrocław University of Science and Technology, 50-370 Wroclaw, Poland; miroslaw.sopel@pwr.edu.pl

**Keywords:** extracorporeal shock wave therapy, neuromodulation, neuroregeneration, recovery, spasticity, spinal cord injury, narrative review

## Abstract

Background: Injury of the spinal cord causes motor and sensory dysfunction as well as pathological reflexes, leading to paraplegia or tetraplegia. The sequelae of traumatic spinal cord injury (SCI) are a significant burden and impact on healthcare systems. Despite constant progress in medicine, traumatic SCI still remains irreversible. To date, no satisfying treatment that can enable neuronal regeneration and recovery of function at the damaged level has been found. Hundreds of experiments have been conducted on various possibilities of influencing spinal regeneration; some of them have yielded promising results, but unfortunately, the successes obtained in experimental animals have not translated into humans. Methods: This narrative review article presents the application of extracorporeal shock wave therapy (eSWT) in patients with SCI. The article has been divided into parts: 1) use of extracorporeal shock wave therapy for regeneration of the spinal cord after traumatic spinal cord injury; 2) application of extracorporeal shock wave therapy in spasticity after spinal cord injury. In both cases, the hypotheses of possible mechanisms of action will be described. Results and conclusions: A small number of clinical trials have demonstrated the potential of eSWT to influence the regeneration of the spine, as an innovative, safe, and cost-effective treatment option for patients with SCI. Some reports have shown that eSWT can improve spasticity, walking ability, urological function, quality of life, and independence in daily life.

## 1. Introduction

Despite constant progress in medicine, traumatic spinal cord injury (SCI) still remains irreversible. The sequelae of the spinal cord are motor and sensory dysfunction as well as pathological reflexes, leading to paraplegia or tetraplegia, imposing a significant burden and impact on healthcare systems. For diagnostic purposes, the International Standards for Neurologic Classification of Spinal Cord Injury, endorsed by the American Spinal Injury Association (ASIA) and the International Spinal Cord Society (ISCoS), are commonly utilized [1].

To date, no satisfying treatment that can provide effective neuronal regeneration and motor recovery has been found. The most significant pathophysiological mechanisms of SCI are disruption of microcirculation and the resulting secondary injury occur as a consequence of a series of biochemical and pathological processes [2]. Hundreds of experiments have been conducted on various possibilities of influencing spinal regeneration, some of yielding promising results; unfortunately, the successes obtained in experimental animals have not translated into humans.

The goal of drug interventions is to enhance microcirculation, which can positively influence the microenvironment and support SCI-related recovery. In 2024, Wang and Bai detailed pharmacological strategies aimed at improving microcirculation after traumatic SCI. They classified the drugs into seven categories: neurotrophic factor-related drugs, synthetic chemical compounds and biological agents, corticosteroids, inhibitors or antagonists of endogenous vasoactive substances, matrix metalloproteinase (MMP)-related drugs, human immunoglobulin G (HIGG), carbon monoxide (CO) donors, and other pharmacological agents. Clinical tests indicated that these drugs show promise for enhancing functional recovery following SCI, reducing blood–spinal cord barrier (BSCB) damage, improving intimal protection, and promoting repair and regeneration. By enhancing blood flow in the spinal cord, these drugs can modify the injury microenvironment and facilitate spinal cord tissue repair [3].

Comprehensive rehabilitation plays a crucial role for SCI patients—this should be focused on achieving optimal independence in activities of daily living (ADL) and improving the ability to walk [4]. Currently, new innovative methods of walking recovery based on robots have appeared; one of them is robotic-assisted gait training [5,6].

For more than three decades, experimental research has increasingly explored the therapeutic potential of stem cells in SCI, largely due to their capacity to differentiate into neural cells and secrete neurotrophic factors. Bonosi et al. [2] provided a comprehensive overview summarizing the current state of research, challenges, and future directions for stem cell therapy in SCI models. The findings are generally optimistic, indicating that stem-cell-based therapies offer benefits for SCI treatment by stimulating neuroregeneration and neuroprotection. However, several safety concerns have been highlighted, including immunotoxicity, immunogenicity, and carcinogenicity, frequently discussed in preclinical studies. Common obstacles such as limited cell survival and integration have been noted, affected by variables like cell number, treatment timing, and transplantation strategies. Additionally, genetic stability, generational consistency, and storage safety of stem cells are crucial considerations. For clinical application, understanding the mechanisms of action and biological properties is essential. Challenges in registered clinical trials include small sample sizes, limited oversight, and varying quality, which hinder the development of stem cell therapies. Establishing standard protocols is difficult due to the heterogeneity of injury types and levels, treatment timings, and cell quantities. Bonosi et al. [2] concluded that integrating genetic engineering technologies, cell conjugation, combination therapies with neuroprotective factors, trophic factors, biomaterials, and rehabilitation could enhance the therapeutic efficacy of stem cells across diverse patient populations.

In a recent review article, Rahmadian et al. [7] described biomaterials used to treat this disease and techniques used to create nanostructured biomaterials. They noted that nanomedicine innovations in SCI management hold promise in bridging the gap. In another recent review article, Tian et al. [8] presented the latest advances and challenges in the treatment of SCI. The authors explored the future of SCI treatment, focusing on strategies to bridge the gap between preclinical research and clinical practice. They recommended that clinical trials prioritize interdisciplinary dialogue and collaboration to determine the best combinatorial approaches for enhancing therapeutic outcomes in SCI patients. Youseffard et al. [9], based on a systematic review of 14 studies and 17 separate animal experiments, found that the self-assembling peptide could potentially aid in the recovery of motor function after brain injury through axonal regeneration help locomotion recovery after SCI due to axon regeneration.

It seems that the future of neuronal regeneration lies in the use of 3D bioprinted scaffolds. Szymoniuk et al. [10] performed a systematic review of experimental studies on this topic. Eleven preclinical rat model trials of SCI were identified; six studies utilized 3D bioprinted scaffolds with stem cells, two combined 3D bioprinted scaffolds with growth factors, and three employed stand-alone 3D bioprinted scaffolds, each demonstrating varying levels of bias risk. Significant functional improvements were noted in all included studies compared to control groups. Functional recovery aligned with histological changes observed at the injury site. The authors concluded that 3D bioprinted scaffolds could be a viable therapeutic option for SCI treatment, though additional evidence from other experimental SCI models is required before clinical application.

This narrative review article has been divided into parts: (1) use of the extracorporeal shock wave therapy early after traumatic SCI, and (2) application of extracorporeal shock wave therapy in spasticity post-SCI. In conducting this narrative review, we performed a targeted search of the scientific literature using databases such as PubMed, Web of Science, and PEDro. The search focused on studies published between 2020 and 2024 that specifically investigated the use of eSWT in the context of SCI. Only full-text articles published in English were considered for inclusion, with an emphasis on clinical and experimental trials. The selection criteria for this review emphasized clinical studies that contributed to understanding the role of eSWT in spinal cord regeneration and spasticity management. Preclinical studies or studies that did not focus on spinal cord injury or spasticity were excluded. Although the review did not follow a systematic protocol, the synthesis was designed to provide a comprehensive overview by integrating findings from relevant clinical trials within the designated time frame.

## 2. Use of the Extracorporeal Shock Wave Therapy after Traumatic Spinal Cord Injury

In the literature, physical methods used to regenerate a damaged spinal cord are rare. Neuromodulation techniques for the irreversibly injured spinal cord are better known, including electrical spinal cord stimulation (SCS), which can be administered epidurally or via implanted electrodes; transcutaneous spinal cord stimulation (TSCS); vagus nerve stimulation (VNS); electroacupuncture; direct transcutaneous current stimulation (tDCS); etc. Despite the literature confirming the independent benefits of such stimulation, there are reports confirming that it may be more effective in restoring motor and autonomic functions in combination with physical exercise or stem cell transplantation [11,12,13,14,15,16].

In a recent review article, Liu et al. [17] discussed the potential of artificial hibernation medicine in safeguarding nerves and organs post-SCI. Mild hypothermia, an artificial hibernation technique, has shown tentative clinical benefits for SCI. Recent studies indicate that artificial hibernation technologies could offer therapeutic benefits for nerve damage after SCI by reducing inflammation, providing immunosuppression, enhancing oxidative defense, and possibly offering central protection.

Extracorporeal shockwave therapy (eSWT) is a type of mechanotherapy that generates peak pressures approximately 1000 times greater than ultrasound method. Initially, eSWT was used as a non-invasive, outpatient alternative to surgery for various joint and tendon disorders. SWT was first used in the urology clinic at the University of Munich in 1980 for lithotripsy of human kidney stones and was approved by the US Food and Drug Administration in 1984 [18]. Currently, eSWT is a treatment method that uses strong acoustic pulses, which are most often used in the treatment of kidney stones and in physiotherapy to reduce pain, increase metabolism at the cellular level, revascularize and regain normal muscle tone after various diseases (mainly in diseases of the musculoskeletal system, such as frozen shoulder, tennis elbow, degenerative knee joint, tendinopathies, low back pain, sports injuries) and orthopedics [19,20]. It has also been used as effective modality in the treatment of wounds, male erectile rejuvenation, urological conditions, and even in neurogenic heterotopic ossification [21].

It should be noted that eSWT promotes biological and neural effects through a combination of mechanotransduction, cavitation, and biochemical signaling. Mechanotransduction triggers cellular responses by converting mechanical forces into biochemical signals, while cavitation stimulates micro-damage that initiates tissue repair. eSWT enhances angiogenesis, improving blood flow and tissue perfusion by upregulating growth factors like VEGF. It also has anti-inflammatory effects, reducing pro-inflammatory cytokines (e.g., IL-1, TNF-α) and promoting tissue healing. In terms of neural actions, eSWT increases the expression of neurotrophic factors like BDNF, activates the MAPK pathway, and supports neuroregeneration and neuroplasticity. Additionally, shock waves modulate pain pathways by disrupting nociceptors and promoting the release of endorphins, leading to analgesic effects. Overall, eSWT supports tissue regeneration, neuroprotection, and pain relief, making it an effective therapy for both musculoskeletal and neural injuries [19,20].

Based on the biophysical terms, eSWT can be categorized into focused (feSWT) and radial (reSWT) types. The waves are generated using electrohydraulic, electromagnetic, or piezoelectric methods and then directed to a focal tissue zone. Focused shock waves (feSW) are characterized by high pressures exceeding 1000 bar (100 MPa), extremely short rise times (<10 ns), brief durations (<10 ms), and broad frequency spectra (16–20 MHz). In contrast, radial extracorporeal shock waves (reSW) lack the features of true shock waves, such as short rise times, high peak pressures, and nonlinearity. eSWT is thought to produce two significant physical effects on tissues: mechanotransduction and cavitation. These effects facilitate the penetration of shock waves into tissues, triggering physiological responses at both the molecular and tissue levels, which result in positive biological outcomes such as tissue regeneration and repair, angiogenesis, pain relief, metabolic activation, and anti-inflammatory effects, ultimately leading to favorable therapeutic results [22]. Considering the mechanisms of action of eSWT, Simplicio et al. [23] indicates vascularization, protein biosynthesis, cell proliferation, neuroprotection and chondroprotection. d’Angelo et al. [24] characterize eSWT as a form of mechanotransduction and foresee exciting and beneficial applications in regenerative medicine, tissue engineering, and cell therapies.

Guo et al. [25] published a review on the possibility of regeneration and repair of neurological disorders using the eSWT method. They noted that recent animal studies and clinical trials have demonstrated the potential of eSWT as an innovative, safe and cost-effective option for the treatment of neurological disorders and diseases of the peripheral and central nervous system. The effectiveness of eSWT treatment is influenced by several key parameters: air pressure (measured in bars), energy flux density (EFD, measured in mJ/mm^2^), number of pulses, and frequency (measured in Hz). EFD represents the energy intensity of shock waves per unit area. It has been suggested that ESWT be categorized into low energy (<0.08 mJ/mm^2^), medium energy (<0.28 mJ/mm^2^), and high energy (<0.60 mJ/mm^2^) based on the EFD value.

In a study on the treatment of chronic SCI with eSWT, Lee et al. [26] demonstrated that behavioral performance in rats significantly improved when stem cell therapy was augmented with eSWT. Additionally, focused eSWT (feSWT) was administered at three distinct energy intensities (level 1: 0.01 mJ/mm^2^; level 2: 0.04 mJ/mm^2^; and level 3: 0.11 mJ/mm^2^), with 1000 pulses targeted to the spinal cords of the subjects. Histological analysis revealed that no neurological complications arose from applying feSWT at these energy settings [26]. Other research corroborated these findings, indicating that eSWT administered to rats with spinal cord injuries minimized neural tissue damage, enhanced neuroprotective outcomes, stimulated BDNF production, upregulated serum-derived miRNA expression, and improved motor abilities, all without causing adverse effects [27,28,29,30].

Leister et al. [31] launched a prospective, multicenter, randomized, placebo-controlled study to assess the impact of eSWT on motor and sensory recovery in patients with acute spinal cord injuries (SCI) within six months post-injury. The study aims to enroll 82 patients in its initial phase, involving 15 hospitals and employing a two-arm, three-stage adaptive trial design. Concentrated eSWT, with an energy flux density ranging from 0.1 to 0.19 mJ/mm^2^ and a frequency of 2–5 Hz, will be applied once at the injury site, five spinal segments above and below, as well as on the plantar surfaces of both feet, all within the first 48 h following the injury. The primary outcome measure is the degree of improvement in motor and sensory functions at six months. Secondary outcome measures include routine blood chemistry assessments, spasticity scores, walking ability, urinary function, quality of life, and the level of independence in daily activities. Researchers believe eSWT promotes neural tissue regeneration through various biochemical and cellular events, reducing neuronal loss. They hypothesize that eSWT could enhance acute SCI treatment in future clinical applications.

## 3. The Application of Extracorporeal Shock Wave Therapy in Spasticity after Spinal Cord Injury

Spasticity is a frequent and debilitating consequence of upper motor neuron injury, making diagnosis and treatment crucial to prevent contractures, reduce pain, and enhance functional recovery. The incidence of spasticity after SCI has not been precisely estimated. Dragojlovic et al. [32] conducted a retrospective study on the incidence of spasticity during the first admission to inpatient rehabilitation in 285 patients with acute stroke, traumatic brain injury, and traumatic brain injury. In SCI, the incidence of spasticity was 48% on admission and 46% on discharge.

Based on a 5-year follow-up of 350 patients with an average age of 46 ± 19 years and an average time from SCI to hospital discharge of 108 ± 95 days (range 1–728 days), Mills et al. [33] created the first predictive model for the development of problem spasticity. The factors include age and initial Glasgow Coma Scale (GCS) score at the time of injury; whether the patient was admitted to a rehabilitation facility before hospital discharge or discharged directly from an acute hospital to the community; the use of anti-spasticity medications at discharge; and neurological condition at discharge (including neurologic level, motor level, AIS grade, and changes in AIS motor score), spasm frequency, and patient-reported impacts of pain on activity, sleep, and quality of life.

It turned out that no recommendations regarding electrical stimulation parameters in SCI spasticity could be made precisely due to the wide variability of the methodology. Future studies that are better designed and at the highest methodological level are needed. Several studies have investigated the application of repetitive transcranial magnetic stimulation (rTMS) and transcranial direct current stimulation (tDCS) in the context of spinal cord injury (SCI). In a systematic review and meta-analysis, Chen et al. [34] assessed the effectiveness of non-invasive brain stimulation (NIBS) in improving motor function after brain injury. The study reviewed 14 randomized controlled trials comprising 225 participants, with nine studies utilizing repetitive transcranial magnetic stimulation (rTMS) and five employing transcranial direct current stimulation (tDCS). The meta-analysis revealed that NIBS significantly improved lower extremity strength, balance, and spasticity reduction. However, the findings showed that improvements in upper limb motor function were not statistically significant when compared to control groups, and there was no significant difference in functional mobility between the NIBS and sham groups. Despite marginal *p* values and considerable heterogeneity, the authors concluded that NIBS exerts a beneficial effect on lower limb motor function in SCI patients. They emphasized the need for further high-quality clinical trials to confirm these findings and refine stimulation parameters for optimal use by clinicians in their practice.

Adeel et al. [35] explored the impact of paired stimulation using specific waveforms on cortical and spinal plasticity in individuals with chronic spinal cord injury (SCI) who had sustained their injuries for at least one year. The study involved three therapeutic interventions administered in random order for 4 to 20 min, followed by 30 min of cycling. These interventions included a control condition, repetitive transcranial magnetic stimulation (rTMS) at 20 Hz paired with trans-spinal direct current stimulation (tsDCS), and intermittent theta burst stimulation (iTBS) combined with tsDCS, with a one-week washout period between treatments. Resting motor threshold (RMT) was assessed using transcranial magnetic stimulation (TMS), with 90% of the RMT value used as the stimulation intensity. Additionally, the Hoffman (H) reflex was evaluated by stimulating the tibial nerve at the popliteal fossa. Primary variables, including RMT, motor evoked potential (MEP) latency, MEP peak-to-peak amplitude, and H-reflex latency, were measured before and after the interventions. Secondary variables consisted of the Lower Extremity Motor Scale (LEMS) and the Modified Ashworth Spasticity Scale (MAS). The results demonstrated significant improvements in MEP latency, MEP amplitude, and LEMS scores when using either the rTMS-iTBS/tsDCS or rTMS-20 Hz/tsDCS protocols (*p* < 0.050) compared to the control condition. Although other measures, such as RMT, H-reflex latency, and MAS scores, showed some changes, they did not reach statistical significance. The authors concluded that rTMS-iTBS/tsDCS was just as effective as conventional rTMS-20 Hz/tsDCS in promoting neuroplasticity in individuals with chronic SCI.

Only a few reports presented the utility of eSWT in the spasticity management after SCI. Comino-Suárez et al. [36] published a case study and literature review on the use of eSWT in the treatment of plantar flexor spasticity in SCI. The authors noted that approximately 65–78% of patients with SCI exhibit symptoms of spasticity. The purpose of their study was to evaluate the tolerability and immediate effects of reSWT on plantar flexor spasticity in an 18-year-old male with incomplete spinal cord injury (SCI) following five reSWT sessions. The results showed that passive range of motion (A-PROM) increased by 15 degrees at T1 and by 25 degrees at T2, in comparison to the baseline measurement at T0SWT on plantar flexor spasticity in an 18-year-old male with incomplete spinal cord injury (SCI) following five reSWT sessions. The results showed that passive range of motion (A-PROM) increased by 15 degrees at T1 and by 25 degrees at T2, in comparison to the baseline measurement at T0. The passive drag force on ankle dorsiflexion at low speed decreased in the gastrocnemius and soleus muscles, and in the gastrocnemius muscle at high speed, with minimal change in the soleus muscle. The authors concluded that reSWT, combined with conventional therapy, is well tolerated and effectively improves A-PROM and passive resistance force in ankle dorsiflexion in the short term. They recommend further randomized, controlled trials with longer follow-up to confirm these results in SCI patients.

## 4. Discussion

As can be seen from the above review, there are many methods of spinal cord regeneration; unfortunately, the promising results do not translate to humans. There are many hypotheses trying to explain the mechanisms of possible spine regeneration. Pharmacological treatments focused on microcirculation can enhance the microenvironment and aid recovery from SCI. These include drugs derived from neurotrophic factors, synthetic chemicals and biological substances, inhibitors or antagonists of endogenous vasoactive substances, corticosteroids, matrix metallopeptidases (MMP) inhibitors, carbon monoxide (CO) donors, human immunoglobulin G (HIGG), and other pharmaceutical agents.

The potential of stem cells in the episodes of SCI is thought to stem from their capacity to differentiate into neural cells and secrete neurotrophic factors. Numerous researchers suggest that employing genetic engineering technologies, cell conjugation, and combination therapies with neuroprotective factors, biomaterials, trophic factors, and rehabilitation techniques could enhance the healing efficacy of stem cells in diverse SCI patients.

It seems that the future belongs to the use of nanotechnology, artificial hibernation techniques, self-assembling peptide, 3D bioprinted scaffolds and extracorporeal shock wave therapy (eSWT). The newest one—eSWT—is a previously known form of mechanotherapy mainly from use in urology and musculoskeletal diseases. It works through mechanotransduction and cavitation triggering beneficial biological processes such as angiogenesis, tissue repair, pain reduction, anti-inflammatory effects, and metabolic activation, ultimately resulting in positive clinical outcomes. Due to the lack of clinical reports, future research is needed to precisely determine the optimal parameters of eSWT: air pressure, energy flux density, number of pulses and frequency.

Gollmann-Tepeköylü et al. [37] reported on an experimental study in mice, demonstrating that eSWT exhibits strong regenerative properties for bone fractures, wounds, myocardial ischemia, and SCI by activating the innate immune receptor 3 (TLR3). TLR3 signaling has been identified as a crucial factor for spinal cord regeneration, as shown in zebrafish models. In human spinal cord cultures, activation of TLR3 in combination with eSWT promoted neuronal sprouting and improved neuronal survival. In wild-type (WT) mice with SCI, eSWT prevented neuronal degeneration, enhanced IL6-dependent recruitment and differentiation of neural progenitor cells, improved motor function, and reduced lesion size. However, these beneficial effects were absent in TLR3 −/− mice, highlighting the essential role of TLR3 in neuroprotection and spinal cord repair. These findings suggest that stimulating TLR3 through eSWT could represent a promising therapeutic strategy for promoting regeneration and recovery in SCI.

The pioneer of electrical stimulation in spasticity was Hufschmidt, who, in 1966, described the reduction in spasticity by rhythmic electric shock rectangular electric current of spastic muscles, and then their weakened antagonists during relaxation of spastic muscles. Hufschmidt looked for a mechanism reduction in spasticity of the central component (in addition to the peripheral effect) [38]. In the Faculty of Electrical Engineering, University of Ljubljana (Slovenia), various methods of spinal cord electrical stimulation were originated, notably functional electrical stimulation (FES), enabling walking in paraplegic persons.

In 1970, Jusic and Fronjek [39] published about their own successive modification of Hufschidt’s tonolysis in 32 spastic patients. Franek et al. [40] also described their own methods of electrostimulation in spastic paraplegic patients. Bekhet et al. [41] noted that recommending specific stimulation parameters is challenging due to significant variability in the study design, methodological issues and heterogeneity of the studied articles. Their review included 23 clinical and preclinical trials involving a total of 389 participants. Neuromuscular electrical stimulation (NMES) and functional electrical stimulation (FES) demonstrated a 45–60% reduction in spasticity, decreased electromyography activity, and improved range of motion following SCI. The optimal stimulation parameters identified were a frequency of 20–30 Hz, a pulse duration of 300–350 μs, and a current amplitude exceeding 100 mA. These findings suggest that NMES/FES is an effective rehabilitation approach for managing spasticity post-SCI. They included in a systematic review 23 clinical and experimental trials with 389 individuals appearing spasticity after SCI. Neuromuscular electrical stimulation or FES reduced spasticity up to 45–60%, decreased electromyography activity, and increased the range of motion. The optimal stimulation parameters identified were a frequency of 20–30 Hz, a pulse duration of 300–350 μs, and a current amplitude greater than 100 mA. The authors concluded that neuromuscular electrical stimulation and/or functional electrical stimulation serves as an effective rehabilitation strategy for spasticity management [41]. However, future studies with better design and higher methodological standards are needed.

Massey et al. [42] performed a meta-analysis to assess the impact of different electrical stimulation methods on spasticity in SCI. They examined both neurophysiological and clinical outcomes across various techniques, including transcutaneous spinal cord stimulation (TSCS), transcutaneous electrical nerve stimulation (TENS), and FES for cycling and gait. The primary measures included the Modified Ashworth Scale (MAS), Ashworth Scale (AS), Penn Spasm Frequency Scale (PSFS) and Pendulum Test. Additionally, secondary effects were the Hoffman (H) reflex, posterior-root reflexes (PRRs), and motor-evoked potentials (MEPs). It occurred that muscle activation was not essential for achieving a reduction in spasticity; there was no correlation between clinical and neurophysiological outcomes.

These are few reports on using neuromodulatory methods such as transcranial direct current stimulation (tDCS) and repetitive transcranial magnetic stimulation (rTMS) in SCI. Further high-quality clinical trials are recommended to either validate or challenge the use of NIBS and to refine its stimulation parameters for optimal application in clinical practice.

There are few reports on the application of eSWT in spasticity after SCI. Further RCTs with long-term observations are needed to validate the findings. There are many more reports on the application of eSWT in poststroke spasticity, multiple sclerosis, and cerebral palsy.

In 2021, Polish researchers [43], based on 20 years of experience, described the current state of knowledge on the clinical and methodological aspects of eSWT in the treatment of post-stroke spasticity. A total of 21 reports were found, including 432 patients after stroke—10 reports regarding the treatment of upper limb spasticity (249 patients) and 11 reports regarding the treatment of lower limb spasticity (183 patients). Reviewers focused their attention on the clinical and methodological aspects of this issue. Many types of devices are produced: electromagnetic, electrohydraulic, piezoelectric or pneumatic, using concentrated SWT or radial SWT. Different SWT parameters are used, including the number of impulses, pressure, frequency and energy, as well as varying the number of sessions and application sites—such as the spastic abdominal muscle or the distal or proximal muscle attachment. Various timings of this intervention were observed: in some reports, eSWT was initiated one month after the onset of stroke; in others, it was initiated 198 months after the onset of stroke. Various inclusion criteria were used. Dozens of primary and secondary outcome measures were used. Several studies lacked a control group. Nevertheless, eSWT has been shown to effectively reduce muscle tension in individuals with spastic limbs post-stroke and is considered safe without unwanted side effects. The exact mechanism by which eSWT affects muscles with spasticity remains unknown. Currently, there are no standardized parameters for eSWT in treating post-stroke spasticity, including intensity, frequency, location, and the number of sessions. Therefore, it is concluded that further high-quality research is necessary to establish the recommended parameters for muscle stimulation using eSWT [43].

Of the articles published in 2023, three seem interesting. Afzal et al. [44] presented a systematic review and meta-analysis of the effects of eSWT on spasticity, gait, and quality of life in post-stroke lower limb spasticity. A total of five studies with 389 participants were considered for inclusion. In the experimental group, compared to the control group, a beneficial effect of eSWT on spasticity, mobility (assessed with the TUG test) and motor function of the lower limbs was observed. However, there is uncertainty about its effectiveness on walking performance.

Lee and Kim [45] systematically reviewed 33 papers from 2003 to 2023 on pain level, balance control and post-stroke spasticity. Various shock wave generation methods and their applications have shown positive therapeutic effects in stroke patient rehabilitation. These include improvements in balance, pain reduction, decreased spasticity, enhanced muscle control, and strengthened functional activities of the upper and lower limbs. The effectiveness of eSWT can vary based on the patient’s condition, the method of application, and the treatment area.

Based on a meta-analysis of 13 studies including 677 participants, Ou Yang et al. [46] found that spasticity decreased after eSWT throughout a three-months follow-up period. Limb functionality improved significantly in the short follow-up period and could persist for at least 2 weeks. Patients who experienced stroke less than 45 months prior may benefit from eSWT, showing improved limb functioning over long-term observation periods.

In a comprehensive review of 18 systematic reviews, Khan et al. [47] evaluated the evidence supporting various non-pharmacological interventions for treating spasticity across different neurological conditions. They found ‘moderate’ evidence supporting the use of neuromuscular stimulation and acupuncture as adjunct therapies to conventional care (including pharmacological and rehabilitation treatments) for post-stroke individuals. The quality of evidence was rated as ‘low’ for spasticity-focused rehabilitation interventions, such as induced movement therapy, stretching, dynamic elbow splinting, and occupational therapy in stroke and other neurological conditions. Similarly, the evidence was low for eSWT in brain injury, tDCS for conditions affecting jumping, TMS and TENS in other neurological disorders, physical activity programs and rTMS in multiple sclerosis patients, vibration therapy in SCI, and stretching in other neurological conditions. For all other interventions, the evidence was deemed inconclusive. The authors emphasized the need for further research employing appropriate study designs, varied methodologies, and cost evaluations to thoroughly assess the effectiveness of these interventions [47].

Several articles in the literature attempt to explain the potential theoretical mechanism of eSWT’s analgesic effects. Based on their review of the literature, Chamberlain and Colborne [48] highlighted the complexity of trying to explain the effects of eSWT at the cellular and molecular levels. Their research highlighted several key players in promoting repair, including runt-related transcription factor 2 (RUNX2), bone morphogenetic proteins (BMPs), vascular endothelial growth factor (VEGF), and the mitogen-activated protein kinase (MAPK) cascade. The MAPK pathway encompasses protein kinase cascades activated by genotoxic stress and growth factors, including chemotherapeutic agents.

The electrophysiological mechanisms of the antispastic action of eSWT still remain unclear. It is important to note that, to the best of the current knowledge, there are no existing observations of the resting bioelectrical activity of treated spastic muscles using surface electromyography (sEMG) or the superficial temperature distribution of these muscles using infrared thermometry (IRT) imaging. Given these findings, further research is warranted on specialized and non-invasive tools such as sEMG and IRT imaging, which enable precise measurement of significant phenomena and factors related to the potential antispastic mechanisms of eSWT [49].

While current studies suggest that eSWT may have beneficial effects on muscle tone and strength in patients with SCI, precise mechanisms and quantitative data on these outcomes are not well-documented. Future research should aim to address this gap by incorporating standardized measurements, such as the Modified Ashworth Scale (MAS) for muscle tone and dynamometry for muscle strength, both before and after eSWT treatment. These measurements would provide valuable insights into the specific impact of eSWT on muscle function, allowing for a more comprehensive understanding of its therapeutic benefits.

There is also limited information available regarding the long-term neurological effects of eSWT in patients with SCI. Most existing studies have focused on short- and medium-term outcomes, leaving questions about the sustainability of neurological improvements and the possibility of late-onset side effects. Long-term follow-up studies are needed to thoroughly evaluate the durability of eSWT’s benefits, particularly in terms of sustained improvements in motor and sensory function, as well as to monitor for any delayed adverse effects. Such studies will be crucial in establishing the long-term efficacy and safety of eSWT as a treatment option for SCI.

It is crucial to maintain thorough documentation of any adverse events that occur during or after eSWT treatment. While the therapy is generally considered safe, especially in the short term, any unexpected outcomes—such as pain, discomfort, or bone-related complications—should be promptly reported and carefully documented. Although existing studies on the application of eSWT have not identified significant risks of bone damage or fractures, the potential for such risks cannot be entirely dismissed, particularly in SCI patients who may have compromised bone integrity due to immobility or disuse osteoporosis. We recommend that clinicians exercise caution when administering prolonged eSWT therapy and consider radiographic assessments before and after treatment sessions to monitor bone integrity. Additionally, any adverse events, including potential bone-related complications, should be promptly reported and documented to further inform the safety profile of eSWT. This precautionary approach will help ensure patient safety and minimize any potential adverse effects on bone structure.

### Strengths and Limitations

This narrative review provides an in-depth and comprehensive synthesis of the current literature on the application of eSWT in SCI. Narrative reviews offer the flexibility to cover a broad range of topics, including studies with heterogeneous designs and methodologies, which is particularly valuable in emerging fields such as eSWT. By allowing for more interpretative analysis and the exploration of complex trends, this review highlights potential mechanisms, therapeutic benefits, and areas for future research that may not yet be ready for systematic review. Furthermore, narrative reviews allow researchers to identify gaps in knowledge and suggest innovative perspectives for advancing the field.

However, as a narrative review, this work does not adhere to the rigid, systematic selection process employed by systematic reviews and meta-analyses. Consequently, there is a risk that some studies may be overlooked, particularly those not easily accessible or published in less widely known journals. Additionally, the inclusion of studies with varying levels of quality may introduce bias. Despite these limitations, we have endeavored to include studies that are relevant and of sufficient quality to ensure a reliable and balanced perspective on the topic. Future systematic reviews and meta-analyses will be essential for confirming and expanding upon the conclusions presented here. Moreover, it is important to note, however, that many of these studies did not adjust for potential confounding factors such as age, overall health, concurrent treatments, or injury severity. As such, while the reported outcomes are promising, the potential influence of these confounders cannot be entirely excluded. Future research should incorporate more robust study designs that control for these variables to ensure that the observed effects can be more confidently attributed to eSWT itself. Although this review focuses on the motor and sensory effects of eSWT in SCI patients, the impact of eSWT on autonomic functions such as bladder and bowel control has not been thoroughly investigated. Given the significant influence of these functions on quality of life, future research should examine the potential effects of eSWT on bladder and bowel control in this patient population.

## 5. Conclusions

Hundreds of experiments have been carried out on various possibilities of influencing the regeneration of the spine, some of them giving promising results; unfortunately, the successes obtained on experimental animals have not translated into humans. This narrative review article explores the use of eSWT for SCI patients. The article is divided into two parts: (1) the use of eSWT in the regeneration of the spinal cord after traumatic SCI and (2) the use of eSWT in spasticity after SCI. A small number of clinical trials have demonstrated the potential of eSWT as an innovative, safe and cost-effective treatment option for patients with SCI. Some reports have shown that eSWT can improve spasticity, walking ability, urological function, quality of life and independence in daily life. In both cases, one can only hypothesize possible mechanisms of action.

Moreover, the integration of eSWT with other rehabilitation therapies, such as physiotherapy and occupational therapy, represents a promising area for future research. While eSWT has demonstrated beneficial effects on spasticity and muscle tone, the potential for synergistic effects when combined with conventional rehabilitation therapies has not yet been fully explored. Future studies should aim to investigate the efficacy of combined therapy protocols, assessing whether the integration of eSWT with other modalities can enhance outcomes of SCI patients in terms of functional recovery, mobility, and quality of life. Also, future studies should incorporate functional independence measures, such as the Barthel Index, to evaluate how eSWT affects patients’ ability to perform daily activities independently. This would provide a more comprehensive understanding of the therapy’s overall impact on quality of life and long-term rehabilitation outcomes.

Currently, there are no established standardized parameters for the use of eSWT in treating spastic muscles following spinal cord injury, including specific guidelines for intensity, frequency, application sites, or the number of treatments required. Further research is needed to assess the effect of eSWT on SCI using appropriate study designs, method time and intensity, and associated costs.

To sum up, studies investigating the use of eSWT in SCI patients commonly use feSWT rather than reSWT with EFD typically ranging between 0.04 and 0.19 mJ/mm^2^, which is classified as low to medium energy. The number of pulses administered per session usually ranges from 1000 to 3000 pulses, delivered at frequencies of 3–5 Hz. Treatment protocols often involve 1 to 3 sessions per week, with the therapy continuing for a duration of 3 to 8 weeks, depending on the severity of the injury and patient needs. These parameters have shown promise in promoting neuroregeneration, reducing spasticity, and improving overall functional outcomes in SCI patients. However, further research is required to standardize these protocols for particular SCI consequences and ensure optimal results for individual patients.
